# Supplementary material for: Dynamic transcriptomic profiles of zebrafish gills in response to zinc supplementation
Source: BMC Genomics. 2010 Oct 11;11:553. doi: 10.1186/1471-2164-11-553 (PMC3091702; doi:10.1186/1471-2164-11-553)
Supplement: Additional file 2 — Interactive Direct Interaction Network representing the molecular interactions between zinc, copper, iron, calcium and proteins encoded by transcripts changed by zinc supplementation. Mini web-site containing index.html and hyperlinked pages in subdirectory describing a Direct Interaction Network automatically generated based on curated interactions contained within the proprietary PathwayArchitect database. Ovals represent proteins and the circles symbolize metal ions. Objects are coloured by their abundance in zebrafish at the time-point they were significantly different from the control is a scale from -4 fold (dark green) to +4 fold (dark red). Where significant differences were found at more than one time-point, the colour overlay shows expression at the first instance. Dark blue squares denote 'binding', and light blue squares 'expression'; green squares stand for 'regulation', green diamonds for 'metabolism', and green circles for 'promoter binding'. Arrow heads indicate directionality of the interaction where annotated. All nodes and edges can be further interrogated by selecting the relative area of the image. [file 1471-2164-11-553-S2.zip › PathwayArchitect Zn xs DIN/956643.html]

# REGULATION:

|  |  |
| --- | --- |
| Type | REGULATION |
| Effect | Positive |


---

|  |  |
| --- | --- |
| Score | 0 |


---

|  |  |
| --- | --- |
| Reference Count | 5 |


---

|  |  |
| --- | --- |
| Mechanism | Unknown |


---

|  |  |
| --- | --- |
| Reference:0 || Sentence | "Here we report cloning of the human homologs to IscU and NifU, iron-binding proteins that play a critical role in Fe-S cluster assembly in bacteria." |
| PMID | 11060020 |
| Year | 2000 |
| Species | Human |
| Journal | EMBO J |
| RefScore | 1 |
| Source | PArchNLP |
  |
|  |  |


---

|  |  |
| --- | --- |
 Reference:1 || Sentence | "Moreover, the high levels of Fe found in the substantia nigra of PD patients may perhaps be explained by free Cu binding to iron binding protein-1 (IBP-1), causing it to malfunction and preventing it from detaching itself from the transferrin receptor (TfR) inhibition gene, resulting in expression of TfR even when the cell has plenty of Fe." |
| PMID | 11425282 |
| Year | 2001 |
| Species | Human |
| Journal | Med Hypotheses |
| RefScore | 1 |
| Source | PArchNLP |
  ||  |  |


---

|  |  |
| --- | --- |
 Reference:2 || Sentence | "Assembly of the Fe-S clusters is mediated by the iron-sulphate cluster assembly (ISC) machinery consisting of some ten proteins." |
| PMID | 10916152 |
| Year | 2000 |
| Species | Human |
| Journal | Trends Biochem Sci |
| RefScore | 0 |
| Source | PArchNLP |
  ||  |  |


---

|  |  |
| --- | --- |
 Reference:3 || Sentence | "This was to determine if the uptake of transferrin-bound iron (Tf-Fe) and NTB-Fe uptake is mediated by a common iron-carrier." |
| PMID | 9303500 |
| Year | 1997 |
| Species | Human |
| Journal | Hepatology |
| RefScore | 1 |
| Source | PArchNLP |
  ||  |  |


---

|  |  |
| --- | --- |
 Reference:4 || Sentence | "At a lower Tf-Fe concentration of (0.125 micromol/L) when uptake of iron is TR-mediated, a 10-fold molar excess of iron citrate had no effect on Tf-Fe uptake by HuH7 TR antisense and sense cells." |
| PMID | 9303500 |
| Year | 1997 |
| Species | Human |
| Journal | Hepatology |
| RefScore | 2 |
| Source | PArchNLP |
  |


---

|  |  |
| --- | --- |
